# *Parachironomus* Lenz from China and Japan (Diptera, Chironomidae)

**DOI:** 10.3897/zookeys.494.6837

**Published:** 2015-04-06

**Authors:** Chun-Cai Yan, Jiao Yan, Li Jiang, Qin Guo, Ting Liu, Xin-yu Ge, Xin-Hua Wang, Bao-ping Pan

**Affiliations:** 1 Tianjin Key Laboratory of Animal and Plant Resistance,Tianjin Normal University, Tianjin, 300387, China; 2 College of Life Sciences, Nankai University, Tianjin 300071, China

**Keywords:** Chironomidae, *Parachironomus*, new species, new combinations, new synonyms, key

## Abstract

Members of the genus *Parachironomus* Lenz known from China and Japan are revised, and a key to their male adults is given. *Parachironomus poyangensis* **sp. n.** is described in this life stage. *Parachironomus frequens* (Johannsen) and *Parachironomus monochromus* (van der Wulp) are recorded from China for the first time, thus are redescribed from Chinese specimens. *Parachironomus kamaabeus* Sasa & Tanaka and *Parachironomus toneabeus* Sasa & Tanaka are new junior synonyms of *Parachironomus frequens*. Three Chinese or Japanese species formerly placed in *Parachironomus* are transferred to other genera, resulting in the new combinations *Cryptochironomus inafegeus* (Sasa, Kitami & Suzuki), Demicryptochironomus (Irmakia) lobus (Yan, Sæther, Jin & Wang), and *Microchironomus lacteipennis* (Kieffer). *Chironomus sauteri* Kieffer, *Parachironomus kisobilobalis* Sasa & Kondo and *Parachironomus kuramaexpandus* Sasa are removed from *Parachironomus*; the last of these three denotes a valid species of uncertain generic placement, the first two are *nomina dubia*.

## Introduction

The name *Parachironomus* was proposed by Lenz (1921) for a genus concept based on larval and pupal characters. Edwards (1929) gave the first brief diagnosis for male imagines. Townes (1945) treated Nearctic species which are now considered as *Parachironomus* in “Harnischia (Harnischia)”, but his classification and nomenclature of Chironomini were very different from those in use today (e.g. Cranston et al. 1989; Makarchenko et al. 2006; Sæther and Spies 2013). However, Townes’ designation of *Chironomus cryptotomus* Kieffer, 1915 as the type of *Parachironomus* has been accepted as formally valid, even though the taxonomic identity of that species is uncertain (*Chironomus cryptotomus* Kieffer is a nomen dubium). Among the known genera in the *Harnischia* group, the genus *Parachironomus* is closer to *Demicryptochironomus* Lenz (1941); it is distinguished from the later in having long superior volsella with 2–3 distal setae usually arising from distinct pits, inferior volsella with blunt or pointed caudal projection, while in *Demicryptochironomus* usually no the setal pits of superior volsella and inferior volsella reduced or absent.

Freeman and Cranston (1980) synonymized *Kribiocryptus* Kieffer, 1921 and *Nilomyia* Kieffer, 1921 under *Parachironomus* Lenz, 1921. However, Spies and Sæther (2004) showed that any name available from Kieffer (1921b, published in June) would take precedence over any name available from Lenz (1921, October). In this situation, using *Parachironomus* as a valid name could comply with the current rules of nomenclature (ICZN 1999) only if a special ICZN ruling were to effect an exemption from priority in this case, or if *Kribiocryptus* and *Nilomyia* are no longer treated as synonymous with *Parachironomus*. The latter classification has been adopted by Sæther and Spies (2013), and is followed here.

Lehmann (1970) revised 17 European species and gave a generic diagnosis and key to species. Spies et al. (1994) revised members of the genus from the Neotropical Region, and modified the generic definition. Later, *Parachironomus supparilis* (Edwards, 1931) was split in three species: *Parachironomus longistilus* Paggi, 1977, *Parachironomus supparilis* (Edwards), and *Parachironomus valdiviensis* Spies (Spies 2008). Spies (2000) studied the Palaearctic *Parachironomus monochromus* (van der Wulp) and the Holarctic *Parachironomus tenuicaudatus* (Malloch) in all stages, and presented a provisional key to adult males from Nearctic Region.

Hashimoto et al. (1981) placed six species from Thailand in *Parachironomus*: *Parachironomus apicalis* (Kieffer), *Parachironomus calopunctus* Hashimoto, *Parachironomus truncatus* Hashimoto, *Parachironomus nakhonphanomensis* Hashimoto, *Parachironomus tener* (Kieffer), and *Parachironomus trisetifer* Hashimoto). However, if the partially incomplete published descriptions are correct, then all of these forms except possibly *Parachironomus calopunctus* obviously fall outside of the current diagnosis of *Parachironomus*. Moreover, the corresponding material is either lost or inaccessible. Under these circumstances, no species proposed in Hashimoto et al. (1981) is treated as valid in *Parachironomus* in the present work. Maheshwari and Agarwal (1993) published a *Parachironomus agraensis* from India, but insufficient description and inaccessible type material (M. Spies, pers. comm.) render this yet another nomen dubium in Chironomini. Kikuchi and Sasa (1990) described a *Parachironomus tobaquartus* from Indonesia, but several hypopygial features of that species clearly rule out placement in *Parachironomus*. *Cryptochironomus lacteipennis* Kieffer and *Chironomus sauteri* Kieffer were listed in *Parachironomus* by Sublette and Sublette (1973), along with *Chironomus primitivus* Johannsen. However, the assignment of genus names used in that work does not match that of today (for example, “*Parachironomus*” included *Microchironomus* Kieffer). Moreover, the original description of *Chironomus sauteri* treats the adult female only; thus the name could not possibly be interpreted by Sublette and Sublette or any recent author without examination of the syntypes (at SDEI, Müncheberg, Germany).

Makarchenko et al. (2005) listed nine species from the Russian Far East: *Parachironomus biannulatus* (Staeger), *Parachironomus forceps* (Townes), *Parachironomus frequens* (Johannsen), *Parachironomus gracilior* (Kieffer) [sub *Parachironomus arcuatus* (Goetghebuer)]. *Parachironomus monochromus* (van der Wulp), *Parachironomus paradigitalis* Brundin, *Parachironomus parilis* (Walker), *Parachironomus pseudovarus* Zorina), and *Parachironomus vitiosus* (Goetghebuer); Zorina in Makarchenko et al. (2006) keyed eight of these species but omitted *Parachironomus forceps*.

From 1985–2001, Sasa and various co-authors, and Kobayashi and Suzuki (1999) recorded 11 species from Japan: *Parachironomus gracilior* (Kieffer) [sub *Parachironomus arcuatus* (Goetghebuer)], *Parachironomus harunasecundus* Sasa, *Parachironomus inafegeus* Sasa, Kitami & Suzuki, *Parachironomus inageheus* Sasa, Kitami & Suzuki, *Parachironomus kamaabeus* Sasa & Tanaka, *Parachironomus kisobilobalis* Sasa & Kondo, *Parachironomus kuramaexpandus* Sasa, *Parachironomus monochromus* (van der Wulp), *Parachironomus tamanipparai* (Sasa), *Parachironomus taishoabeus* Sasa & Tanaka, and *Parachironomus toneabeus* Sasa & Tanaka). Yamamoto (2010) keyed 7 species from Japan: *Parachironomus acutus*, *Parachironomus gracilior* [sub *Parachironomus arcuatus*], *Parachironomus kisobilobalis*, *Parachironomus kuramaexpandus*, *Parachironomus monochromus*, *Parachironomus swammerdami* (Kruseman) (which might also be *Parachironomus mauricii* (Kruseman) or an unnamed species), and *Parachironomus tamanipparai* (this belongs to *Saetheria* Jackson; M. Spies, pers. comm.). Based on the present examinations, only fpur or five true *Parachironomus* species appear to be known from Japan: *Parachironomus frequens* (Johannsen), *Parachironomus gracilior* (Kieffer), *Parachironomus monochromus* (van der Wulp), and *Parachironomus swammerdami* (Kruseman); *Parachironomus acutus* (Goetghebuer) is only provisionally placed in the genus at this time.

Wang et al. (1977) recorded *Cryptochironomus arcuatus* Goetghebuer, 1919 (= *Parachironomus gracilior* (Kieffer, 1918)) and *Cryptochironomus primitivus* Johannsen from Hubei Province, China. Wang (2000) listed both species in the genus *Parachironomus*. However, *Cryptochironomus primitivus* Johannsen has been treated as a synonym of *Microchironomus tener* (Kieffer) since Sæther (1977). Wang and Ji (2003) recorded *Parachironomus arcuatus* (= *Parachironomus gracilior*) in Oriental China (Fujian Province). In addition, Wang (2000) recorded *Parachironomus varus* (Goetghebuer) from Tianjin, but upon rechecking the specimen we are correcting that identification to *Parachironomus gracilior*. *Parachironomus lobus* Yan, Sæther, Jin & Wang was recorded by Yan et al. (2008b) from Hainan Province. According to an examination of type specimens by M. Spies, the species should be placed in the genus *Demicryptochironomus*.

Based on the known descriptions and material from China and Japan, the genus is reviewed, and one new species is described in the adult male stage. A key to adult males from China and Japan is provided.

## Material and methods

The material examined was mounted on slides following the procedure outlined by Sæther (1969). The morphological nomenclature follows Sæther (1980) with the additions and corrections given by Sæther (1990). Measurements are given as ranges followed by the mean when more than three specimens were measured, followed by the number measured (n) in parentheses.

Type material studied is housed in the following institutions: Wang collection, Department of Biology, Life Science College, Nankai University, Tianjin, China (BDN); Sasa collection, National Science Museum, Tokyo, Japan (NSM).

### Provisional key to adult males of *Parachironomus* from China and Japan

**Table:** 

1	Tergite IX with shoulder-like caudal margin	**2**
–	Tergite IX with triangle caudal margin	**4**
2	Gonostylus with distinct expansion basally; anal point parallel-sided	***Parachironomus acutus* (Goetghebuer)**
–	Gonostylus without distinct expansion basally or parallel-sided; anal point not parallel-sided	**3**
3	Anal point with constriction proximal of apical swelling; gonostylus with constriction in middle	***Parachironomus frequens* (Johannsen)**
–	Anal point pointed; gonostylus parallel-sided	***Parachironomus swammerdami* (Kruseman)**
4	Gonostylus with distinct widening in distal 1/3; superior volsella slightly curved, swollen distally	***Parachironomus monochromus* (van der Wulp)**
–	Gonostylus widened basally or parallel-sided; superior volsella straightly, finger-like	**5**
5	Frontal tubercles absent; mid and hind tibiae each with 1 spur; gonostylus parallel-sided	***Parachironomus poyangensis* sp. n.**
–	Frontal tubercles present; mid and hind tibiae each with 2 spurs; gonostylus widened basally	***Parachironomus gracilior* (Kieffer)**

## Species in *Parachironomus*

### Parachironomus frequens

Taxon classificationAnimaliaDipteraChironomidae

(Johannsen)

Figs 1–4

Chironomus frequens Johannsen, 1905: 230. – Malloch (1915: 452).
Chironomus (Cryptochironomus) lhoneuxi Goetghebuer, 1921b: 168.
Cryptochironomus longiforceps Kieffer, 1921d: 66.
Harnischia (Harnischia) frequens (Johannsen). – Townes (1945: 155).
Parachironomus frequens (Johannsen). – Lehmann (1970: 143); Pinder (1978: 132).
Parachironomus toneabeus Sasa & Tanaka, 1999: 38, **syn. n.**
Parachironomus kamaabeus Sasa & Tanaka, 2001: 45, **syn. n.**

#### Material examined.

CHINA: 1 male, Hebei Province, Zunhua City, Dongling, Longmenkou Reservoir, 7. vii. 2001, Y. Guo; 1 male, Yunnan Province, Kunming City, Yiliang County, 2. vi. 1996, X. Wang; 1 male, Xizang Autonomous Region, Nyalam County, Zhangmu Town, 2400 m, a. s. l., 16. viii. 1987, light trap, C. Deng.

JAPAN: Holotype of *Parachironomus kamaabeus* Sasa & Tanaka, 2001 (No. 391: 45), male, Gunma Prefecture, Tone River, Taisho Bridge, light trap, 1. vii. 1999. -Paratype of *Parachironomus kamaabeus* Sasa & Tanaka, 2001 (No. 391: 47), male, Gunma Prefecture, Tone River, Taisho Bridge, light trap, 3. vii. 1999.

#### Diagnostic characters.

The species is distinguished by the following combination of characters: mid and hind legs with dark brown rings, tergite IX with shoulder-like caudal margin; basal half of anal point with lateral setae, superior volsella finger-like.

#### Redescription

(Chinese specimens). Male imago (n=3, unless otherwise stated). Total length 4.15–4.70, 4.46 mm. Wing length 1.98–2.54, 2.33 mm. Total length / wing length 1.78–2.10, 1.93. Wing length / length of profemur 2.20–2.47, 2.37.

**Figures 1–4.**
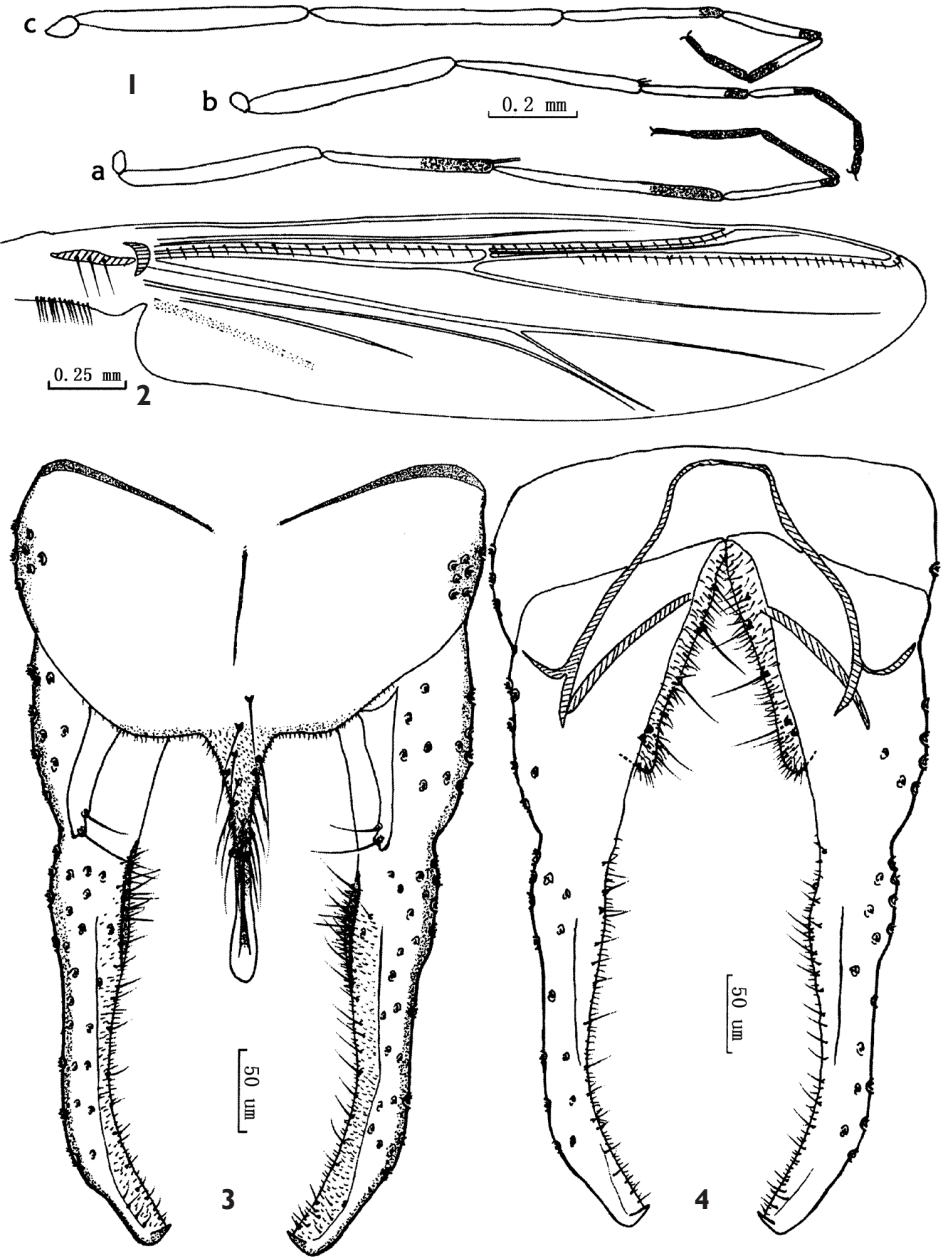
*Parachironomus frequens* (Johannsen), Chinese specimens. **1** Legs **a** front leg **b** mid leg **c** hind leg **2** Wing **3** Dorsal view of hypopygium **4** Ventral view of hypopygium.

*Coloration*. Thorax yellowish green to yellowish brown. Femora and tibiae of front legs yellowish green with distal 1/3 of tibiae and tarsi I dark brown, tarsi II with distal dark brown rings, tarsi III–V dark brown; femora and tibiae of mid and hind legs yellowish green, tarsi I, II of mid legs and tarsi I–III of hind legs pale with distal dark brown rings, tarsi III–V of mid legs and tarsi IV, V of hind legs completely dark brown (Fig. 1). Abdomen yellowish green to yellowish brown.

*Head*. AR 2.66–2.86, 2.79. Terminal flagellomere 850–1030, 960 mm long. Frontal tubercles absent. Temporal setae 16–17, 17, including 3–4, 4 inner verticals, 7–9, 8 outer verticals and 5–6, 5 postorbitals. Clypeus with 19–26, 22 setae. Tentorium 100–150, 133 mm long, 40–50, 47 mm wide. Palpomere lengths (in mm): 37–55, 47; 55–68, 59; 145–220, 184; 160–213, 181; 178–338, 255. Length ratio 5^th^ /3^rd^ palpomere 0.95–1.71, 1.40.

*Thorax*. Antepronotals 3–5 (2), acrostichals 5–9 (2), dorsocentrals 10–12 (2), prealars 5–9 (2). Scutellum with 18–20 (2) setae.

*Wing* (Fig. 2). VR 1.16–1.18, 1.17. R with 20–25, 22 setae, R_1_ with 20–21, 21 setae, R_4+5_ with 22–26, 24 setae. Brachiolum with 3–4, 3 setae. Squama with 13 setae.

*Legs*. Front tibia with 3 subapical setae, 110 (1), 138–140 (2) and 150 (2) µm long; spurs of mid tibia 28–48, 36 and 33–50, 41 µm long, comb with 40–56, 47 teeth, 10–15, 12 µm long; spurs of hind tibia 33–55, 42 and 42–75, 56 µm long, comb with 56–68, 62 teeth, 10–15, 12 µm long. Tarsus 1 of mid leg with 22 sensilla chaetica, hind legs without sensilla chaetica. Lengths (in µm) and proportions of legs as in Table 1.

**Table 1.** Lengths (µm) and proportions of adult male legs in *Parachironomus frequens* (Johannsen), (n=3).

	fe	ti	ta_1_	ta_2_	ta_3_	ta_4_	ta_5_	LR
p_1_	900–1030, 980	800–960, 893	1080–1250 (2)	550–650 (2)	420–500 (2)	330–390 (2)	150–200 (2)	1.30–1.35 (2)
p_2_	1030–1150, 1043	850–1060, 970	460–570, 523	280–350, 323	220–290, 260	150–190, 170	100–130, 116	0.54
p_3_	1060–1300, 1183	1100–1350, 1242	670–820, 757	440–550, 500	350–440, 400	220–270, 243	120–160, 140	0.61

*Hypopygium* (Figs 3, 4). Laterosternite IX with 7–8 (2) setae. Anal tergite bands Y-shaped, fading far apart medially. Tergite IX with shoulder-like margin, bearing 2 setae at base of anal point. Anal point originating from caudal margin of anal tergite, bearing 14–22, 17 lateral setae in basal half, widened at base, constricted medially, slightly swollen apically, 130–155, 143 mm long, 30–35 (2) mm wide at base, 8–12 (2) mm wide in middle, 14–15 (2) mm wide at apex. Transverse sternapodeme 40–50, 46 mm long. Phallapodeme 95–118, 107 mm long. Superior volsella slightly curved, finger-like, slender distally, with an apical seta and a proximal lateral seta, both not arising in conspicuous pits. Inferior volsella with a moderately pointed caudal projection, covered with microtrichia, and reaching beyond anal tergite margin. Gonocoxite 148–175, 158 mm long, with 4–5 (2) strong medial setae. Gonostylus 230–275, 256 mm long with apical hook (2), slightly swollen at base, parallel-sided medially, curved distally, bearing 9–10 (2) setae along the basal inner margin and 12–14 (2) setae along the distal inner margin. HR 0.58–0.64, 0.62; HV 1.64–1.80, 1.74.

#### Distribution.

Holarctic (Sæther and Spies 2013). The species is also known from Japan and China; the record for China is new.

#### Remarks.

Sasa and Tanaka (1999) described *Parachironomus toneabeus* from Japan based on material collected at Kamakura Bridge, Ino River, Gunma Prefecture on 21 August 1998. The sample number was given as “391: 45–47”. Sasa and Tanaka (2001) proposed *Parachironomus kamaabeus* according to material collected at Taisho Bridge, Tone River, Gunma Prefecture on 1 July 1999. However, the number of the specimen is also “391: 45–47”. Based on the type specimens and the original descriptions and figures, we place both *Parachironomus toneabeus* and *Parachironomus kamaabeus* as new junior synonyms of *Parachironomus frequens* (Johannsen), due to distinct matches in leg color, shapes of the anal point, superior volsella and gonostylus, and the shoulder-like tergite IX margin.

### Parachironomus gracilior

Taxon classificationAnimaliaDipteraChironomidae

(Kieffer)

Figs 5–7

Chironomus gracilior Kieffer, 1918: 49. – Goetghebuer (1921a: 42, 163).
Cryptochironomus arcuatus Goetghebuer, 1919: 66. – Wang et al. (1977: 230).
Tendipes (Parachironomus) monotomus (Kieffer). – Kruseman (1933: 193).
Tendipes (Parachironomus) arcuatus (Goetghebuer). – Kruseman (1933: 194).
Tendipes (Cryptochironomus) arcuatus (Goetghebuer). – Goetghebuer (1937 in 1937–1954: 43).
Parachironomus gracilior (Kieffer). – Lenz (1938: 711).
Parachironomus arcuatus (Goetghebuer). – Brundin (1947: 56); Lehmann (1970: 135); Pinder (1978: 132); Sasa (1985: 108); Sasa and Kawai (1987: 20); Sasa (1988: 56; 1989: 23, 849); Sasa and Kikuchi (1995: 102); Sasa (1996: 95; 1998: 30); Wang (2000: 645); Özkan (2002: 186); Wang and Ji (2003: 61); Makarchenko et al. (2005: 410).

#### Material examined.

CHINA: 9 males, Tianjin City, Campus of Nankai University, 9 males, 12, 16. iv. 1985; 15. v. 1985; 20. iv. 1986, X. Wang; 1 male, Tianjin City, Shuanglin Farm, 20. vi. 1985, X. Wang; 1 male, Hebei Province, Qinhuangdao City, 1 male, vi. 1985, X. Wang; Hebei Province, Chicheng County, Yunzhou Reservoir, 21. vii. 2001, sweep net, Y. Guo and Y. Du. 1 male, Neimenggu Autonomous Region, Wuliangsuhai Lake, v. 1982, X. Wang; 1 male, Neimenggu Autonomous Region, Alashan League, Bayin, 30. vii. 1987, X. Wang; 1 male, Liaoning Province, Shenyang City, 27. viii. 1990, J. Wang; 1 male, Jiangxi Province, Poyanghu Lake, Nanjishan, 12. vi. 2004, Sweep net, C. Yan. 1 male, Yunnan Province, Kunming City, Dianchi Lake, 23. v. 1986, X. Wang; 1 male, Yunnan Province, Lijiang City, School of Agriculture Reservoir, 2400 m a.s.l., 28. v. 1996, X. Wang.

#### Diagnostic characters.

The species can be identified by the following combination of characters: anal point moderately narrow; frontal tubercles present; superior volsella bearing two apical setae, short cylindrical, often appearing more or less contracted, and with folds on inner margin; gonostylus with constriction at approximately mid-length.

**Figures 5–7.**
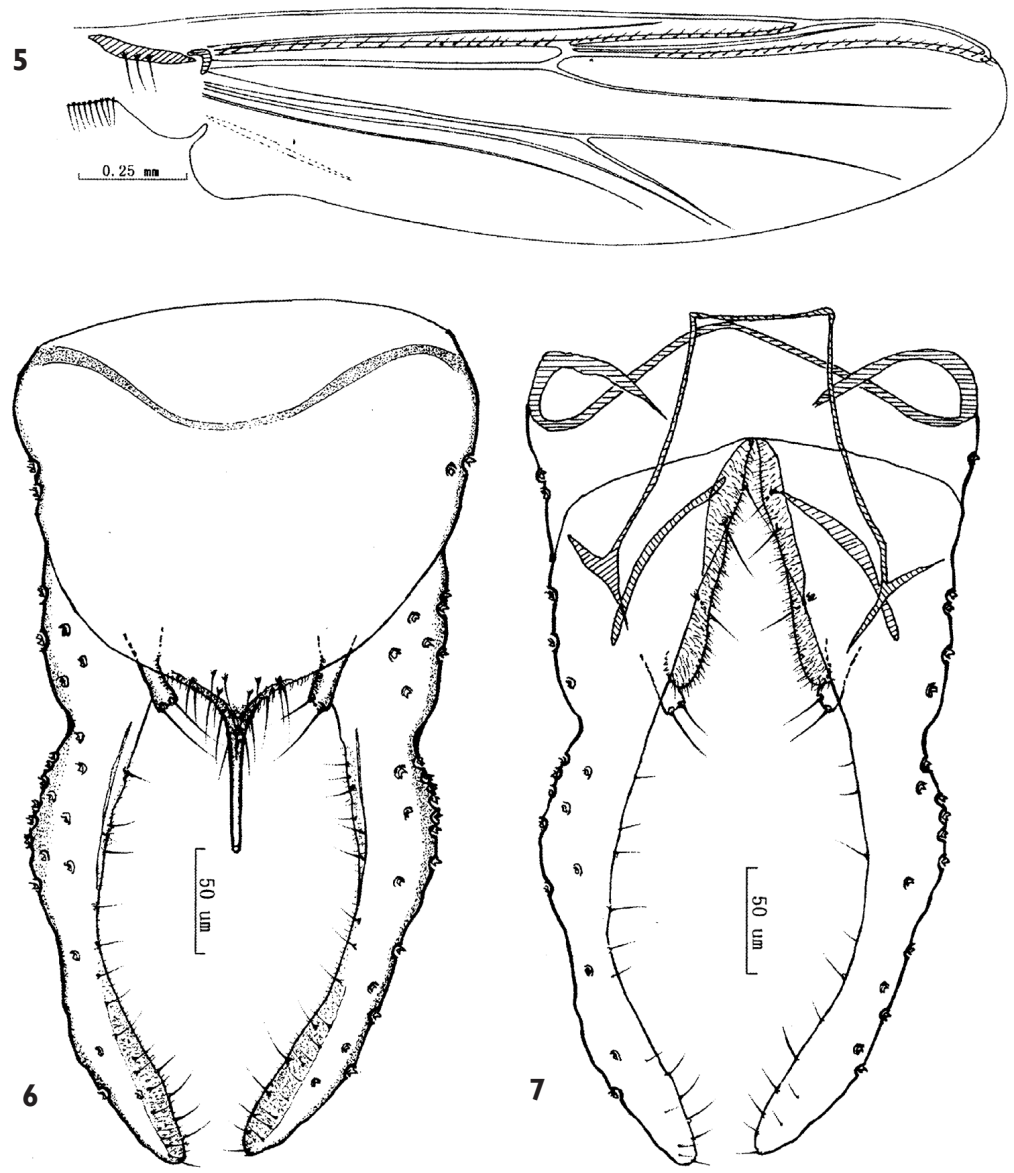
*Parachironomus gracilior* (Kieffer), Chinese specimens. **5** Wing **6** Dorsal view of hypopygium **7** Ventral view of hypopygium.

#### Distribution.

The species is widely distributed in the Palaearctic and extends into the Oriental Region (Sæther and Spies 2013). It occurs in China and Japan.

#### Remarks.

The synonymy between *Parachironomus gracilior* and *Parachironomus arcuatus* was accepted in the past already (e.g. by Goetghebuer 1921a, Lenz 1938); thus we do not present it as a ‘new synonymy’ here. The holotype of *Chironomus gracilior* Kieffer (at SDEI) and non-type specimens identified as *Tendipes monotomus* by Kruseman (1933) have been examined by M. Spies, and found to be conspecific beyond any doubt (M. Spies, pers. comm.).

### Parachironomus monochromus

Taxon classificationAnimaliaDipteraChironomidae

(van der Wulp)

Figs 8–10

Chironomus unicolor van der Wulp, 1859: 5 (primary homonym of *Chironomus unicolor* Walker, 1848).
Chironomus monochromus van der Wulp, 1875: 129 (replacement name for *Chironomus unicolor* van der Wulp).
Chironomus (Cryptochironomus) claviforceps Edwards, 1929: 389.
Tendipes (Parachironomus) monochromus (van der Wulp). – Kruseman (1933: 192).
Tendipes (*Cryptochironomus* gr. *Parachironomus*) *monochromus* (van der Wulp). – Goetghebuer (1937 in 1937–1954: 46).
Parachironomus monochromus (van der Wulp). – Brundin (1947: 55); Lehmann (1970: 146); Pinder (1978: 130); Albu (1980: 131); Langton (1991: 274); Kobayashi and Suzuki (1999: 82); Spies (2000: 126).

#### Material examined.

CHINA: 8 males, Tianjin City, Campus of Nankai University, 8 males, 12, 16. iv. 1985; 20. iv. 1986, X. Wang; 1 male, Hebei Province, Weichang County, Jixielinchang, 15. vii. 2001, sweep net, Y. Guo.

#### Diagnostic characters.

The species is distinguished by the following combination of characters: anal tergite with distinct cluster of enlarged posterodorsal setae; anal point basal section intergrading with anal tergite, distal part strongly angled to ventral; superior volsella without apical or posterolateral projection; inferior volsella with lobe at least to median; gonostylus mostly slender, slightly curved, with distal widening to dorsal peaking around 2/3 of gonostylus length (excerpt from Spies 2000: 129).

#### Redescription

(Chinese specimens). Male imago (n=9, unless otherwise stated). Total length 2.58–3.83, 3.38 mm. Wing length 1.30–1.98, 1.80 (8) mm. Total length/wing length 1.80–1.98, 1.88 (8). Wing length/length of profemur 2.28–2.57, 2.48 (8).

*Coloration*. Thorax yellowish green to dark brown. Front legs with femora yellowish green to dark brown, tibiae and tarsi dark brown except for tarsi I yellowish green in basal 4/5; mid and hind legs yellowish green to yellowish brown except for tarsi V dark brown. Abdomen yellowish green to dark brown.

*Head*. AR 1.86–2.27, 2.12. Terminal flagellomere 540–720, 673 µm long. Frontal tubercles absent (7) or present (2), cone-shaped, 15–22 µm high, 12–22 µm wide at base. Temporal setae 18–22, 20, including 5–7, 6 inner verticals, 7–8, 8 outer verticals, and 5–8, 6 postorbitals. Clypeus with 14–20, 17 (8) setae. Tentorium 100–133, 120 µm long, 18–43, 33 µm wide. Palpomere lengths (in µm): 30–50, 40; 35–58, 51; 103–133, 110; 130–180, 153 (8); 178–220, 201 (8). Length ratio 5^th^/3^rd^ palpomere 1.21–1.73, 1.58 (8).

*Thorax*. Antepronotals 2–5, 3 (8), acrostichals 10–14, 12 (8), dorsocentrals 8–14, 11, prealars 4–6, 5 (8). Scutellum with 6–10, 8 (7) setae.

**Figures 8–10.**
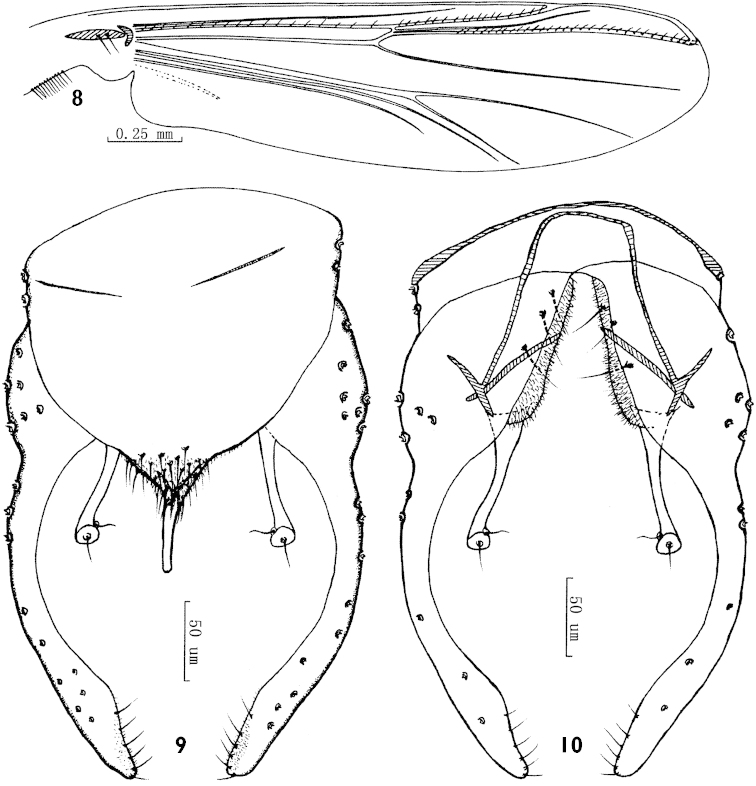
*Parachironomus monochromus* (van der Wulp), Chinese specimens. **8** Wing **9** Dorsal view of hypopygium **10** Ventral view of hypopygium.

*Wing* (Fig. 8). VR 1.11–1.17, 1.15 (8), R with 16–27, 20 (8) setae, R_1_ with 10–17, 13 (8) setae, R_4+5_ with 21–29, 26 (8) setae. Brachiolum with 2–3, 2 (8) setae. Squama with 7–16, 12 (8) setae.

*Legs*. Front tibia with 3 subapical setae, 90–130, 104 (7), 98–133, 118 (6) and 120–138, 126 (3) µm long; spurs of mid tibia 24–33, 28 µm and 28–35, 31 µm long, comb with 30–42, 35 teeth, 10–12, 11 µm long; spurs of hind tibia 26–33, 31 µm and 28–35, 33 µm long, comb with 45–52, 48 teeth, 10–13, 12 µm long. Tarsus 1 of mid leg with 4–7, 6 (8) sensilla chaetica, hind leg without sensilla chaetica. Lengths (in µm) and proportions of legs as in Table 2.

**Table 2.** Lengths (µm) and proportions of adult male legs in *Parachironomus monochromus* (van der Wulp) (n=9).

	fe	ti	ta_1_	ta_2_	ta_3_	ta_4_	ta_5_	LR
p_1_	570–790, 727	420–620, 576	670–930, 863 (6)	340–460, 432 (6)	270–370, 342 (6)	210–270, 247 (6)	110–140, 133 (6)	1.45–1.64, 1.55 (6)
p_2_	550–780, 728	480–710, 640	260–360, 326 (8)	140–310, 198 (8)	100–160, 140 (8)	70–110, 98 (8)	60–90, 85 (8)	0.44–0.54, 0.52 (8)
p_3_	620–890, 818	610–950, 849	410–630, 557 (6)	210–350, 312 (6)	180–270, 247 (6)	110–150, 143 (6)	80–110, 104 (6)	0.66–0.70, 0.67 (6)

*Hypopygium* (Figs 9, 10). Laterosternite IX with 2–3, 2 (8) setae. Anal tergite bands short, fading far apart medially. Tergite IX with 16–30, 21 (8) setae at base of anal point. Anal point 35–55, 48 (7) µm long, its base intergrading with conical tip of anal tergite; distal bare part narrow. Transverse sternapodeme 37–60, 52 (8) µm long. Phallapodeme 60–83, 73 (8) µm long. Superior volsella slightly curved, 70–95, 84 µm long, 13–25, 19 µm wide at base, 6–8, 7 µm wide in middle, 12–17, 15 µm wide at apex, without conspicuous apicolateral projection; median pit smaller than distal distinct pit and positioned a little proximal. Inferior volsella blunt with a low projection to posterior, not pointed, not reaching beyond anal tergite margin, and covered by microtrichia. Gonocoxite 88–118, 107 µm long, with 3–4, 3 strong medial setae. Gonostylus 158–213, 187 µm long, slender, curved and parallel-sided, with distinct expansion in distal 1/3, bearing 4–7, 6 (8) setae along distal inner margin. HR 0.49–0.68, 0.59, HV 1.63–2.01, 1.80.

#### Distribution.

Palaearctic (Spies 2000). It also is recorded from Palaearctic China and Japan. The record for China is new.

### Parachironomus poyangensis sp. n.

Taxon classificationAnimaliaDipteraChironomidae

http://zoobank.org/1E9205DA-EB68-420E-9EFC-7D45A851E307

Figs 11–13

#### Etymology.

Named after the type locality. The species epithet is adjectival for the purposes of nomenclature.

#### Type material.

Holotype male (BDN No. 21987). CHINA: Jiangxi Province, Poyanghu Lake, Nanjishan Natural Conservation area, 12. vi. 2004, sweep net, C. Yan. Paratypes: 2 males, data same as holotype.

#### Diagnostic characters.

The new species is distinguished by the following combination of characters: body size small, thorax and abdomen yellowish green, wing cells without setae, mid and hind tibiae each with single spur, anal point nearly parallel-sided, superior volsella elongate digitiform, without distal swelling or projection, gonostylus nearly straight and of about even circumference throughout.

#### Description.

Male imago (n=3). Total length 2.25–2.32, 2.28 mm. Wing length 1.08–1.11, 1.10 mm. Total length / wing length 2.08–2.09, 2.09. Wing length / length of profemur 2.20–2.30, 2.25.

*Coloration*. Thorax yellowish green. Femora of front legs yellowish green with distal parts brown, tibiae and tarsomeres dark brown; mid and hind legs yellowish green with tarsomeres IV, V dark brown. Abdomen yellowish green.

*Head*. AR 1.76–1.84, 1.80. Terminal flagellomere 450–470, 462 mm long. Frontal tubercles absent. Temporal setae 11–13, 12, including 3 inner verticals, 3–4, 3 outer verticals and 5–6, 5 postorbitals. Clypeus with 15–18, 17 setae. Tentorium 90–95, 92 mm long, 25–26, 25 mm wide. Palpomere lengths (in mm): 25–27, 26; 30–32, 31; 83–85, 84; 98–104, 100; 145–156, 151. Length ratio 5^th^ /3^rd^ palpomere 1.75–1.78, 1.77.

*Thorax*. Antepronotals 3–4, 3; acrostichals 8–9, 9; dorsocentrals 10–12, 11; prealars 3. Scutellum with 5–6, 5 setae.

**Figures 11–13.**
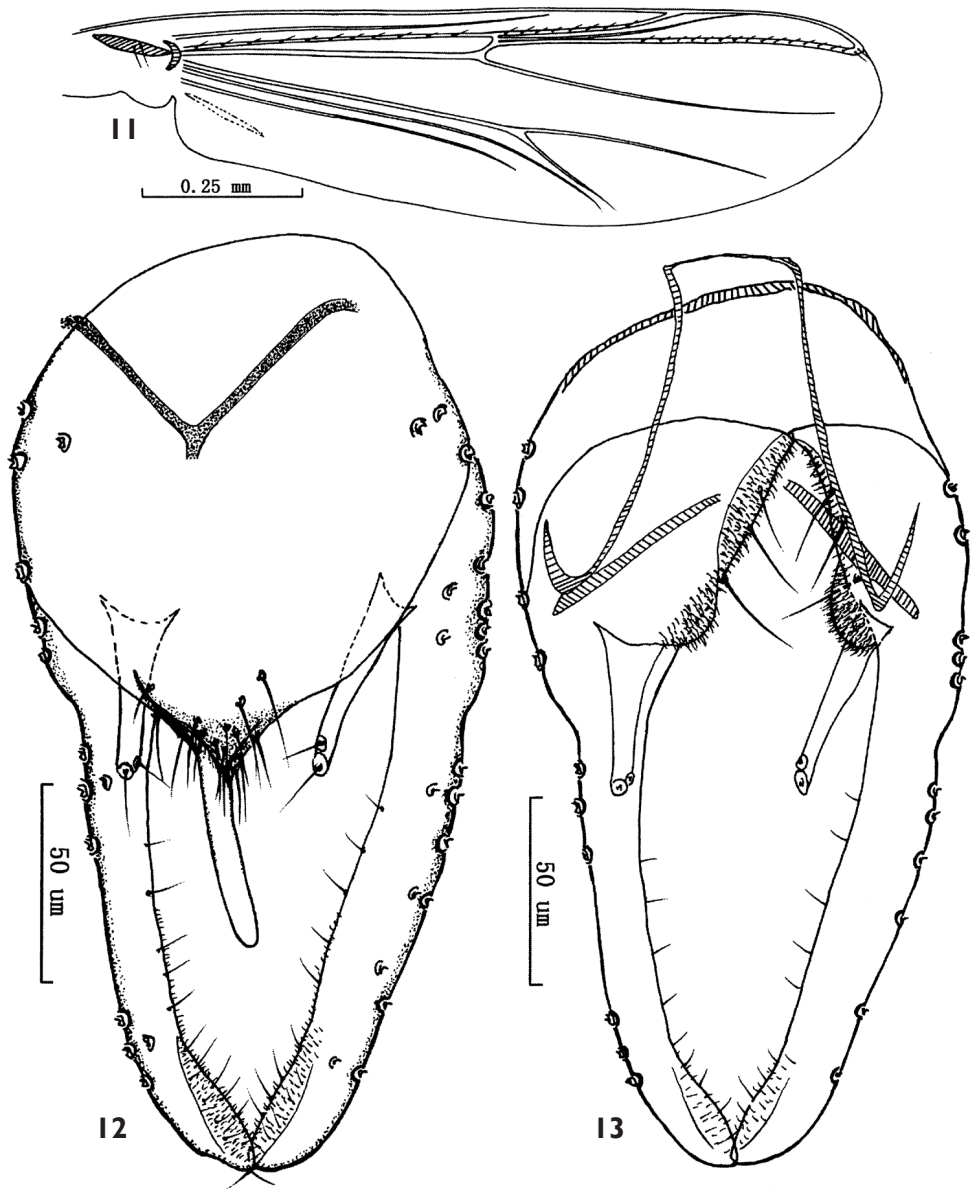
*Parachironomus poyangensis* sp. n. **11** Wing **12** Dorsal view of hypopygium **13** Ventral view of hypopygium.

*Wing* (Fig. 11). VR 1.15–1.17, 1.16. Cell surfaces without setae. R with 11–13, 12 setae, R_1_ with 8–9, 8 setae, R_4+5_ with 17–18, 17 setae. Brachiolum with 2 setae. Squama with 10–12, 11 setae.

*Legs*. Front tibia with 2 subapical setae, 108–110, 109 and 118–130, 126 µm long, mid and hind tibiae each with a single spur, spur of middle tibia 20–22, 21 µm long, comb with 28–30, 31 teeth, 10 µm long; spur of hind tibia 28–30, 29 µm long, comb with 42–46, 44 teeth, 10–12, 11 µm long. Tarsus 1 of mid leg with 6–7, 7 sensilla chaetica, hind leg without sensilla chaetica. Lengths (in µm) and proportions of legs as in Table 3.

**Table 3.** Lengths (µm) and proportions of adult male legs in *Parachironomus poyangensis* sp. n. (n=3).

	fe	ti	ta_1_	ta_2_	ta_3_	ta_4_	ta_5_	LR
p_1_	470–500, 490	310–330, 320	540–560, 550	340-380, 360	230–250, 243	160–180, 166	90–100, 93	1.69–1.74, 1.71
p_2_	440–470, 456	350–380, 363	220–250, 233	120–140, 130	110–130, 120	70–80, 73	50–70, 60	0.63–0.66, 0.65
p_3_	480–530, 503	490–520, 506	340–370, 353	200–220, 210	170–200, 183	100–130, 113	70–80, 73	0.70–0.74, 0.72

*Hypopygium* (Figs 12, 13). Laterosternite IX with 3–4, 3 setae. Anal tergite bands V-shaped. Tergite IX with conical posterior margin, bearing 13–15, 14 setae at base of anal point. Anal point originating from caudal margin of anal tergite, parallel-sided, slightly pointed apically, 50–55, 52 mm long, 6–8, 7 mm wide at base, 4–5, 4 mm wide at apex. Transverse sternapodeme 27–32, 30 mm long. Phallapodeme 45–48, 46 mm long. Superior volsella straight, columnar, distal parts not widened, with an apical seta and a subapical seta, both arising from distinct setal pits. Inferior volsella with a moderately blunt caudal projection, not reaching beyond caudal margin of anal tergite. Gonocoxite 75–80, 77 mm long, with 3–4, 3 strong medial setae. Gonostylus 112–115, 113 mm long, parallel-sided, curved distally, bearing 8–10, 9 setae along distal inner margin. HR 0.65–0.70, 0.67; HV 1.96–2.02, 1.99.

#### Distribution.

The species is known only from the type locality in Oriental China.

### Species removed from *Parachironomus*

#### Microchironomus lacteipennis

Taxon classificationAnimaliaDipteraChironomidae

(Kieffer) comb. n.

Cryptochironomus lacteipennis Kieffer, 1921a: 183.
Parachironomus lacteipennis (Kieffer) – Sublette and Sublette (1973: 405).

##### Remarks.

Kieffer (1921a) described the species in the genus *Cryptochironomus*, which at that time included many species now treated in several separate genera. Sublette and Sublette (1973) placed it in *Parachironomus*. Based on the original description, which describes the inferior volsella as absent, the superior volsella as long and slender, the gonocoxite straight in the proximal 1/3, curved in the distal 2/3, distally attenuated and terminating in an incurved hooklet, *Cryptochironomus lacteipennis* clearly belongs to *Microchironomus* and not to *Parachironomus*. The placement by Sublette and Sublette (1973), and the earlier one in “Tendipes (Parachironomus)” by Kruseman (1939), likely are due to the fact that those authors did not treat *Microchironomus* as a separate genus.

##### Distribution.

The species is recorded from Taiwan Province (Oriental China).

#### Chironomus sauteri

Taxon classificationAnimaliaDipteraChironomidae

Kieffer nomen dubium

Chironomus (Cryptochironomus) sauteri Kieffer, 1921c: 583. – Tokunaga (1940: 301).
Parachironomus sauteri (Kieffer). – Sublette and Sublette (1973: 406).
Cryptochironomus sauteri (Kieffer). – Sasa (1989: 21).

##### Remarks.

Kieffer (1921c) described the species based on females only, and without figures. Tokunaga (1940) described males and females from Taiwan Province, but illustrated only the male superior volsella. Sublette and Sublette (1973) transferred the species to “*Parachironomus*”, but their use of this genus name was different from that of today (i.e., included *Microchironomus* Kieffer). Sasa (1989) examined Tokunaga’s specimens and considered them as belonging to either *Cryptotendipes* or *Microchironomus*, but suggested that the status and placement of *Chironomus sauteri* should be reserved for future clarification. We agree with Sasa’s opinion, but have been unable to examine any of the syntypes; therefore, the species is not included in the present key.

##### Distribution.

The species is known from Taiwan Province (Oriental China).

#### Parachironomus kisobilobalis

Taxon classificationAnimaliaDipteraChironomidae

Sasa & Kondo nomen dubium

Parachironomus kisobilobalis Sasa & Kondo, 1994: 129. – Sasa and Kikuchi (1995: 102); Sasa (1998: 30); Sæther et al. (2000: 190).

##### Material examined.

JAPAN: Holotype of *Parachironomus kisobilobalis* Sasa & Kondo, 1994 (No. A 224: 49), male, Aichi Prefecture, Kiso River in dammed-up middle reach near Nagoya City, “emerged from a sample”, 26. ii. 1992.

##### Remarks.

We have examined the holotype, but it was lacking the thorax, head except for antenna, tarsi of front legs, and half of the hypopygium. As the preserved parts do not suffice for placement of the species, we treat *Parachironomus kisobilobalis* as a *nomen dubium*. In any case, the original description calling the superior volsella “rod-like, with one apical seta and 4 short setae along inner margin” and the inferior volsella “semicircular, with 4 short marginal setae” (Sasa and Kondo 1994: 129; see also Figs 5i–5m) rules out that the species belongs to *Parachironomus*.

##### Distribution.

The species has been recorded only from the type in a Palaearctic part of Japan.

## Discussion

Among the many species previously reported in *Parachironomus* from Japan, only *Parachironomus frequens*, *Parachironomus gracilior*, *Parachironomus monochromus*, *Parachironomus swammerdami*, and possibly *Parachironomus acutus* (Original genus is *Chironomus*) are considered as valid records. Aside from the species treated in the present work, *Parachironomus harunasecundus* Sasa has been transferred to the genus *Demicryptochironomus* (Yan et al. 2008b); *Parachironomus inageheus* Sasa, Kitami & Suzuki, 2001 has been identified as a junior synonym of *Demicryptochironomus ginzancedeus* Sasa & Suzuki (Yan et al. 2008b). *Parachironomus inafegeus* Sasa, Kitami & Suzuki should be transferred to *Cryptochironomus* because of the prominent frontal tubercles, both superior and inferior volsellas carry long setae, the inferior volsella is completely covered by the superior volsella, and the gonostylus is short, rather broad and fused with the gonocoxite. *Parachironomus tamanipparai* (Sasa) was returned to *Paracladopelma* by Yan et al. (2008a), but the holotype (examined by M. Spies) and the published descriptions clearly show it to be a member of *Saetheria* (as recognized earlier, e.g. by Laville and Reiss 1988 and Makarchenko et al. 2006). *Parachironomus taishoabeus* Sasa & Tanaka is a junior synonym of *Saetheria tylus* (Townes) (Kobayashi 2007). *Parachironomus kuramaexpandus* Sasa (examined by M. Spies) probably belongs to an undescribed genus near *Rheomus*, but definitely not to *Parachironomus*.

Based on examination of the holotype and paratype of *Parachironomus lobus* Yan, Sæther, Jin & Wang by M. Spies, *Parachironomus lobus* is related to Demicryptochironomus (Irmakia) latior, but conclusive placement would require knowledge of the immature stages. The end of the superior volsella looks less expanded than in *Demicryptochironomus latior*. For the moment we propose the new combination Demicryptochironomus (Irmakia) lobus and try to find at least the pupa of this species for further comparison with Demicryptochironomus (Irmakia) latior and other congeners.

## Supplementary Material

XML Treatment for Parachironomus frequens

XML Treatment for Parachironomus gracilior

XML Treatment for Parachironomus monochromus

XML Treatment for Parachironomus poyangensis

XML Treatment for Microchironomus lacteipennis

XML Treatment for Chironomus sauteri

XML Treatment for Parachironomus kisobilobalis
